# Conformational Reorganization of the SARS Coronavirus Spike Following Receptor Binding: Implications for Membrane Fusion

**DOI:** 10.1371/journal.pone.0001082

**Published:** 2007-10-24

**Authors:** Daniel R. Beniac, Shauna L. deVarennes, Anton Andonov, Runtao He, Tim F. Booth

**Affiliations:** 1 Viral Diseases Division, National Microbiology Laboratory, Public Health Agency of Canada, Winnipeg, Manitoba, Canada; 2 Department of Medical Microbiology, University of Manitoba, Winnipeg, Manitoba, Canada; Institut Pasteur, France

## Abstract

The SARS coronavirus (SARS-CoV) spike is the largest known viral spike molecule, and shares a similar function with all class 1 viral fusion proteins. Previous structural studies of membrane fusion proteins have largely used crystallography of static molecular fragments, in isolation of their transmembrane domains. In this study we have produced purified, irradiated SARS-CoV virions that retain their morphology, and are fusogenic in cell culture. We used cryo-electron microscopy and image processing to investigate conformational changes that occur in the entire spike of intact virions when they bind to the viral receptor, angiotensin-converting enzyme 2 (ACE2). We have shown that ACE2 binding results in structural changes that appear to be the initial step in viral membrane fusion, and precisely localized the receptor-binding and fusion core domains within the entire spike. Furthermore, our results show that receptor binding and subsequent membrane fusion are distinct steps, and that each spike can bind up to three ACE2 molecules. The SARS-CoV spike provides an ideal model system to study receptor binding and membrane fusion in the native state, employing cryo-electron microscopy and single-particle image analysis.

## Introduction

Viral membrane fusion proteins are responsible both for binding to cellular receptors, and the subsequent fusion of viral and cellular membranes. The paradigm for class I fusion proteins consists of two heptad repeat regions, and a hydrophobic fusion peptide [1]. This motif is present in SARS-CoV [2] and other coronaviruses [3], as well as the hemagglutinin (HA) of influenza [4], gp21 of human T-cell leukemia virus type 1[5], gp41 of HIV[6], GP2 of Ebola virus [7], [8], and the fusion protein of paramyxovirus [9]–[12]. Class I viral fusion proteins can also be divided into two sub-types; those whose fusion mechanism is low pH-dependent such as influenza HA, and those that are pH-independent like the retroviral fusion proteins. In retroviruses, receptor binding itself can trigger fusion, with temperature and redox conditions also influencing the fusion mechanism [13], [14]. The SARS spike appears to be insensitive to redox conditions [15]. For SARS-CoV, it is proposed that the virus is internalized in the cell by endocytosis, and is then exposed to a low pH environment, and it is postulated that proteolytic cleavage between the S1 and S2 domains initiates the membrane fusion process [16]. Although the factors which trigger fusion (endocytosis, pH sensitivity, single receptor vs. primary and co-receptor binding, redox change) differ amongst diverse virus families, all viral fusion proteins are thought to share the same basic fusion mechanism [1], [4], [17]–[20].

A notable feature of the SARS spike is its large mass (∼500 kD per trimer) and striking, club-shaped appearance, from the end-on, this appears like a three-bladed propeller with a radius of 90 Å [21]. Despite the structural differences, the SARS spike performs the same fundamental task in viral entry to the host cell as other class I viral fusion proteins, such as the influenza HA (∼220 kD per trimer). The SARS spike can be subdivided into four structural domains (from N to C terminus); the two large external domains S1 and S2 are largely responsible for receptor binding and membrane fusion, respectively. In many class I viral fusion proteins the analogous peptides are generated by proteolysis of the spike precursor during the maturation process in the host cell, resulting in two peptides with the fusion peptide on the N-terminus of S2. In SARS-CoV the S1/S2 assignment is given based on sequence homology to other viral fusion proteins, however there is no peptide cleavage during maturation. The final two small domains are comprised of a transmembrane domain, and a carboxyterminal cytoplasmic domain, which principally anchor the spike to the viral envelope. The cell-surface molecule ACE2 is the receptor for the SARS spike protein [22] and is a relatively large macromolecule with a diameter of 70 Å. In comparison, the receptor for influenza HA, sialic acid, is much smaller with a 10 Å diameter. The precise mechanisms by which class I viral fusion proteins gain access to the host cell remain unknown. The hypothetical entry process includes several steps that take place in sequence: receptor binding, fusion core re-arrangement, fusion peptide insertion in host cell membrane, refolding of heptad repeats, membrane fusion, and finally viral nucleocapsid transfer [23].

The structures of ACE2 bound to a fragment of the SARS spike containing the receptor-binding domain and the pre- and post-fusion configurations of the fusion core heptad repeats of the spike have been solved to atomic resolution [2], [3], [24]–[26]. In addition, the atomic resolution structures of two neutralizing antibodies bound to the SARS spike receptor-binding domain have been solved [27], [28] showing that blocking of the receptor binding domain, preventing attachment of virions to cell-surface ACE2, is the likely mechanism of virus neutralization by these antibodies. The aim of the present study was to delineate any possible structural changes in the SARS spike that accompanied receptor-binding, and to precisely localize the receptor binding domains (which are only 14% of the total mass of the spike) within the overall structure, employing cryo-EM and image processing of intact virions bound to soluble ACE2. We demonstrate the structural dynamics which accompany spike-receptor binding, which may be involved in triggering membrane fusion.

## Results

**Figure 1:**
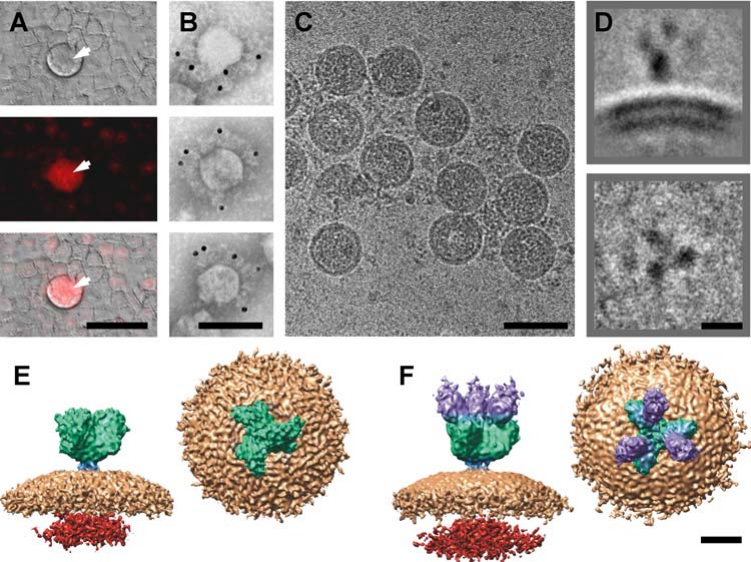
Imaging SARS-CoV, Vero E6 cells, and ACE2. (A) Vero E6 cells stained with propidium iodide. Light microscopy shows a syncytium (white arrow) in the center of the field of view (top), viewed under propidium iodide fluorescence (middle), and an overlay of both images (bottom). (B) Immuno-EM with 10 nm gold confirmed the attachment of ACE2 to the spikes. (C) Cryo-EM field of view of SARS-CoV decorated with ACE2, and select two-dimensional class averages of the SARS spike-ACE2 complex (D). Cryo-EM reconstruction of the SARS spike (E), and SARS spike-ACE2 complex (F), viewed from side and end-on perspectives (left, right). Color scheme: ACE2, violet; spike, green; stalk, blue; envelope, beige; nucleocapsid, red. Scale bars: (A) 50 µm, (B,C) 1000 Å, (D–F) 100 Å.

To study the structure of the spike-ACE2 complex, we infected Vero E6 cells with SARS-CoV and purified supernatant virus by iodoxanol density gradient centrifugation. Previously, we have shown that specimens could be γ-irradiated with a sufficient dose (2Mrad) for viral inactivation, while still preserving protein antigenicity. Inactivation was verified by passage in cell culture and testing by quantitative PCR [29]. Purified γ-irradiated SARS-CoV preparations had a fusogenic activity when added to Vero E6 cells at high multiplicity (∼500–3000 virions per cell), causing the formation of syncytia in the absence of replication or cytopathic effects (Figure 1A). These syncytia had identical morphology to those observed in cytopathic studies of SARS-CoV in tissues and in tissue cultured cells; syncytia have also been observed upon expression of coronavirus S protein, or following addition of cells expressing the S-protein to cells with surface-expressed ACE2. [22], [30]–[34]. The same inactivated γ-irradiated virus preparations were incubated with a human ACE2-human Fc chimeric protein, for analysis of the complex by cryo-EM, 3D image processing, and immuno-electron microscopy (immuno-EM) (Figure 1, Movie S1). The ACE2-Fc construct was selected since it is soluble and dimeric, and it was anticipated that the additional mass was would be beneficial in locating the receptor attached to the spike. Immuno-EM confirmed that recombinant ACE2 protein bound to virions, and that binding affinity was not affected by γ-irradiation (Figure 1B). We did not observe any structural changes associated with prior irradiation. Image averaging further suppressed any possible random radiation induced structural changes.

### Cryo-EM reconstructions of SARS-CoV and ACE2 bound to SARS-CoV

**Figure 2:**
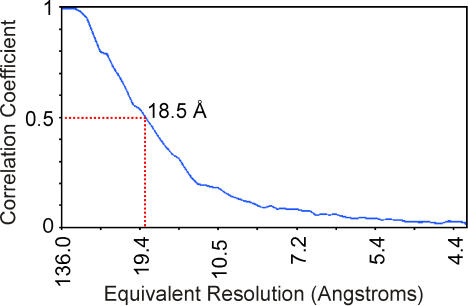
Estimate of resolution of the SARS spike-ACE2 complex. The resolution was estimated by Fourier shell correlation (FSC) between two half reconstructions of the SARS spike-ACE2 complex. The resolution was estimated to be 18.5 Å using the 0.5 FSC criteria (red dotted red line).

**Figure 3:**
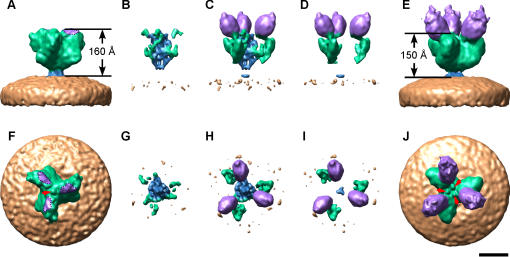
Cryo-EM difference maps. The cryo-EM reconstruction of the SARS spike (A,F) was subtracted from the SARS spike-ACE2 complex (E,J). The positive component attributed to the SARS spike in (B,G) indicates a re-arrangement in the S2 core, and the positive component attributed the SARS-CoV–ACE2 complex shows the addition of ACE2 and an exterior re-arrangement in S1 (D,I). The net difference map is presented in (C,H). The structures are presented from a side perspective (A–E), and end-on perspective (F–J). The arrows in (F) and (J) illustrate the mass reorganization that occurs in the central axis of the spike where one small central blob splits into three nubs. In (A,F) the region on the spike adjacent to ACE2 which corresponds to the receptor binding domain, has been highlighted with a dotted line and is colored purple. The color scheme used for the cryo-EM structure is the same as in Figure 1. Scale bar, 100 Å.

Cryo-EM coupled with 3D single particle image analysis was employed to investigate the binding of ACE2 to the spike of SARS-CoV. Although SARS-CoV is approximately spherical when observed by cryo-EM, both its size and shape vary slightly, and thus it is not amenable to single-particle averaging techniques. However, individual spikes on the surface envelope of the virus do provide a repetitive structure that is ideal for single particle techniques. Spikes on the surface of virus particles are readily imaged by cryo-EM in the frozen-hydrated state (Figure 1C). Three-dimensional image processing was carried out on 11,153 selected spike images taken at various defocus levels, using routine single-particle image processing [35], [36]. The resolution of the final 3D structure was evaluated by a Fourier shell correlation of 0.5 to be 18.5 Å (Figure 2). The structure of the unbound spike was compared with that of the spike-ACE2 complex (Figures 1E, 1F, 3, Movie S2). In both cases the spike portion of the 3D structure is fairly similar, indicating that ACE2 binding does not result in a fundamental structural unfolding of the spike. However, the overall height of the spike was reduced from 160 Å to 150 Å following ACE2 binding. When viewed from the end-on perspective and the spike undergoes a rotation of ∼5° following binding (Movie S2), and the mass at the center of the axis of symmetry on the distal end of the spike redistributes itself from one small central blob to three blobs or nubs (Figure 3 F,J). These redistributions of mass can be further identified in difference maps between the two reconstructions (Figure 3). Figure 3 B,G show that the unbound spike undergoes a decondensation of mass around the central axis (blue) upon ACE2 binding. This region is the putative location of the S2 domain. Whereas, Figure 3 D,I show that the bound spike difference map included both the ACE2 component (purple) and a re-arrangement of the outer edges of the three “blades” of the S1 domain (green).

The precise location of ACE2 binding on the distal end of the spike is centered at 70 Å from the central axis of the spike, with a 30 Å gap between the axis of symmetry and ACE2. One ACE2 molecule was bound to each of the three propeller-like blades of the spike, making a structure 220 Å high (Figure 1F). The density of these ACE2 molecules is high in the 3D density map, indicating a high occupancy of binding sites. In addition, the structure shows that binding of one ACE2 to the spike would not sterically hinder binding of additional ACE2 molecules on the other two propeller-blades of each trimer.

### Docking atomic structures to cryo-EM reconstructions

**Figure 4:**
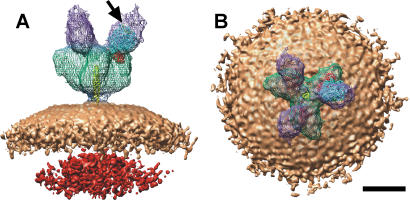
Merging cryo-EM and atomic resolution 3D structures. The atomic resolution structures; PDB ID code, 2AJF (ACE2, blue; receptor-binding domain, red) and PDB ID code, 2FXP (yellow) were docked within the SARS spike-ACE2 complex using SITUS. The SARS spike-ACE2 complex is shown from (A) side and (B) end-on perspectives, the arrow points to the C-terminus of ACE2. The color scheme used for the cryo-EM structure is the same as in Figure 1. Scale bar, 100 Å.

The cryo-EM 3D structures of the spike and the spike-ACE2 complex were combined with the atomic resolution structures of the SARS spike receptor-binding domain- ACE2 complex [24] and the heptad repeat pre- and post-fusion cores [2], [25] to interpret the cryo-EM structure. The receptor-binding domain- ACE2 data were docked with a correlation score of 0.965 using the SITUS software package (Figure 4) [37]. As expected the receptor-binding domain docked to the distal end of the spike with ACE2 filling the extra mass on the spike (shown by the color violet in Figure 4). The empty upper region of the mass is likely composed of the Fc component of the chimeric protein. Although this chimeric molecule is dimeric, only one leg of the ACE-2 is able to bind to each of the three receptor binding domains of the trimer. We anticipate the mass distal to the hinge region to be flexible, and therefore components were blurred out in the averaging process, leaving only a portion of the additional mass in the 3-D map. The C-terminus of the docked ACE2 is in a location consistent with this interpretation. Previously we modeled the putative location of the receptor-binding domain and ACE2 in the cryo-EM structure of the unbound spike using solely the receptor-binding domain atomic resolution data [21]. In the present study, we have shown that our previous prediction of the location of the receptor-binding domain was only partially correct. The actual location of the receptor binding domain and ACE2 are in fact shifted 29 Å towards the 3-fold axis, corresponding to about 40% of the diameter of ACE2. The results from the present study will aid in the precise location of future atomic resolution structure fragments of the SARS spike, as well as in the structural mapping of neutralizing antibody-binding epitopes (which are in progress). The coordinates of the three dimensional reconstruction of the SARS spike (EMD-1423) and the SARS spike-ACE2 complex (EMD-1425) have been deposited in the macromolecular database (http://www.ebi.ac.uk/msd).

## Discussion

Our cryo-EM results demonstrate that there is a structural transition of the spike that occurs upon receptor binding (Figure 3, Movie S2). The overall height of the spike is reduced by 10 Å due to a shift in the mass in the S1 domain (Figure 3 D,I) and the three S1 blades of the spike twist ∼5° along the axis of symmetry. On the distal end of the spike, a small blob feature is replaced by three small nubs which appear radially. (Figure 3 F,J). We propose that this structural re-arrangement represents an initial “priming” of the spike for membrane fusion, which brings the cellular and viral membranes 10 Å closer to each other to facilitate fusion. The coronavirus S protein is known to mediate both virion-cell membrane fusion, and intercellular membrane fusion [22], [30]–[33] and in the present study we have shown that purified inactivated virions are also fusion-competent. We show that the addition of SARS-CoV virions to cultured cells that express ACE-2 can induce fusion, presumably from the fusion activity of spikes on virions bridging neighboring cells. Similar observations on the induction of “fusion-from-without” were made when cells surface-expressing the spike protein were mixed with cells expressing ACE-2 [35]. The trigger that initiates membrane fusion varies amongst class I fusion proteins, with some activated by receptor binding, others by pH, and others by redox conditions. In most proposed models of membrane fusion it is postulated that the S1 domain or analogous receptor binding domains dissociate from the spike during the membrane fusion process. This dynamic process was demonstrated for influenza HA by Kemble *et al.* [38] in their investigation where they engineered intermonomer disulfide bonds between the HA S1 subunits. The result of this was that fusion activity was impaired; however it could be restored under reducing conditions. It is likely that the SARS spike shares a similar mechanism, and the structural changes that we have detected represent the initial step in this process.

By analogy with other class I viral fusion proteins, we anticipate that the fusion core of the SARS spike must undergo similar structural re-arrangements during fusion. The receptor-binding domain is localized in a position on the distal end of the molecule, closer to the 3-fold axis than anticipated, yet still in a position that would not impede these structural re-arrangements. Putative mechanisms by which class I viral fusion proteins achieve membrane fusion have been proposed [1], [4], [17]–[19], but complete structural evidence for the role of intermediate structures in these mechanisms has yet to be obtained. The structural biology of this process has been best characterized for the influenza hemagglutinin, and paramyxovirus fusion (F) protein, for which the pre-fusion and membrane fusion pH structures have been determined by X-ray crystallography [4], [11], [12], [39], [40]. All of the subsequent models for class I viral fusion proteins are based on the structural data of these two fusion proteins. A drawback in all of these models is that they are based on recombinant ectodomains that are not proven to exist as a component in the complete molecule, and they lack both membrane-interacting residues, and lipids [4]. The cryo-EM structures presented in this investigation are of intact SARS spike bound to native virion lipid envelopes. The cryo-EM data are very instructive when atomic resolution fragments are docked within the overall molecule, especially as the entire SARS spike has proven to be a difficult subject for X-ray crystallography, and atomic resolution data exist for only a few fragments of the SARS spike. Our cryo-EM results show that it is possible for the spike to attach to three ACE2 receptors at once; this may serve to hold on to the host membrane like a tripod so as to accurately orientate the fusion core. In addition the 30 Å gap between the axis of symmetry and ACE2 provides sufficient space for fusion core rearrangement. Damico et al., [14] demonstrated that the kinetics of binding of the Rous sarcoma virus envelope protein to its receptor suggested that binding of each spike molecule to multiple receptor monomers occurred. It may therefore be possible that other structurally homologous viral envelope proteins can also bind multiple receptors, and this may be a general adaptation that provides the correct temporal and spatial arrangement to bring about membrane fusion. The observation that the SARS spike could bind three soluble ACE2 receptors provides three possible binding states with one, two or three membrane-bound receptors attached to the spike. With one or two receptors bound, the spike and virus have a wide range of movement possible. Whereas, in the case of three bound receptors the spike and its fusion core will be arranged perpendicular to the cell surface with minimal movement possible. It is interesting to note that binding three receptors is the minimum number of binding events required to achieve this perpendicular orientation in three dimensional space. This observation matches up with the conserved trimeric structures of class I fusion proteins which are common amongst enveloped viruses, thus indicating a possible conserved structural-functional relationship may exist. Another line of reasoning is that oligomerisation favors a better “chance” of successful receptor binding and subsequent insertion of the fusion peptide into the host membrane. According to this model, oligomeric multiples higher then three would be favored, whereas the “tripod” model of receptor binding favors the conserved trimeric structures of class I viral fusion proteins.

In Figure 3 we have we have presented the ACE2–SARS structures as they were solved in this cryo-EM investigation. For other fusion proteins like influenza HA1 and HIV GP120 it has been modeled that the re-arrangements upon membrane fusion are dramatic involving a shedding of the above mentioned domains. In this investigation we have detected structural movement of S1 upon ACE2 binding. This could represent the initial phase of this dramatic process that is followed by fusion core re-arrangement, fusion peptide insertion in the host cell membrane, refolding of heptad repeats, and finally membrane fusion.

Analyzing the structure of the spike-receptor complex is essential to understanding how viruses can adapt to utilize receptors from different species and how they may evolve to gain specificity for new receptor types. RNA viruses have a high rate of mutation and recombination [41]. In SARS coronaviruses, the spike is able to retain specific binding affinity for the ACE2 of more than one host species, and rapid evolution to gain specificity for novel ACE2 species has been demonstrated [42], [43]. The structure and large size of the spike of coronaviruses appears to be an adaptation related to utilization of large host cell-surface molecules such as ACE2 as specific receptors. Amongst the Coronavirus family, specific cell surface receptors for the spike protein are all in the range of 60–110 kD [44]. These large host receptor molecules are in turn functionally constrained and thus conserved across species barriers. In utilizing binding to a large receptor molecule, it may be important that the spike S1 domain also acts as a “spacer arm” holding the receptor far enough away from the three-fold axis of symmetry of the spike S2 domain to permit fusion core re-arrangement and subsequent membrane fusion. Such a property necessitates having a large spike molecule. Moreover, multiple receptor binding may have functional significance, enhancing the binding and entry of viruses. Cross-linking of adjacent host receptor molecules could increase the affinity of the virus for its target cell, as well as improving the kinetics of fusion. The SARS spike-ACE2 complex is an ideal model system for the investigation of class I viral fusion protein dynamics, utilizing cryo-EM to investigate fusion protein binding and structural changes in the native state, within virion membranes that are fusogenic. The presence of a common cell fusion mechanism shared by diverse virus families holds the prospect of developing broad-spectrum anti-virals that target these conserved mechanisms, as well as the potential use of these fusogenic proteins in drug delivery systems.

## Materials and methods

### Cells and viruses

SARS-CoV (Tor3 strain) was inoculated onto Vero E6 cells grown in Dulbecco's modified eagle medium supplemented with 10% foetal bovine serum, 100 U/ml penicillin, 100 µg/ml streptomycin, and 0.22 mg/ml l-glutamine at 37°C with 5% CO_2_. Cell supernatant was harvested (300 ml) with a SARS-CoV TCID_50_ titre of 5×10^7^/ml and virus was pelleted using a Beckman SW32 rotor at 28000 rpm for 90 minutes. The virus pellet was resuspended in PBS buffer, layered onto of a 12–30% iodoxanol gradient (Optiprep) and centrifuged in a SW40 rotor at 38000 rpm for 2.5 hours. Fractions (0.7 ml) were collected from the gradient with an Auto Densi-Flow gradient collector (Labcono) and dialyzed against PBS. SARS-CoV-enriched fractions were checked by SDS-Page and Western blotting, and rendered non-infectious by irradiation in a gamma cell on dry ice with a 2Mrad exposure for 90 minutes. Irradiated specimens were tested for infectivity by inoculation onto Vero E6 cells, and examined for cytopathogenic effects for 10 days, followed by blind passage of the cells and testing for the growth of SARS-CoV by PCR [29].

To test for spike fusogenic activity Vero E6 cells were cultured in 96 well plates (Corning). Once the cells were confluent the medium was removed from each well and 20 µl of gamma irradiated SARS-CoV containing 10^10^ pfu/ml diluted 1∶1 and 1∶9 in culture media. The virus dilutions were incubated with the cells for 1 hour at 37°C. After the incubation the plates were washed three times with fresh culture medium to remove unbound virus. The vero E6 cells were observed by light microscopy 2 and 4 days post treatment for the presence of syncytia. Virus growth was not detected at either dilution of irradiated virus, and no cytopathic effect was observed. The presence of nucleic acid in syncytia was investigated by propidium iodide staining and observation by fluorescence microscopy.

### Immuno-electron microscopy

The human ACE2-Fc (human IgG1) recombinant protein (Adipogen) had an initial concentration of 1 mg/ml in PBS and was diluted 1∶10 in PBS for incubation with SARS-CoV, for the remainder of this document this is referred to as ACE2. 2 µl of SARS-CoV were injected into 4 µl of PBS on a formvar-carbon coated 400-mesh nickel grid, and incubated for 1 minute. All incubations were carried out at 20°C. Grids were then washed in PBS six times for 1 minute, followed by a 10-minute block in PBS-G-BSA (PBS pH7.2, 0.2% glycine, 2% BSA). Grids were then washed in PBS-G (PBS pH7.2, 0.2% glycine) six times for 1 minute, followed by a 1-hour incubation on a 20 µl drop of ACE2. Grids were then washed in PBS-G (PBS pH7.2, 0.2% glycine) six times for 1 minute, followed by a 1-hour incubation with rabbit anti-human ACE2 polyclonal IgG (AdipoGen; diluted 1∶200 in PBS). Grids were then washed in PBS-G (PBS pH7.2, 0.2% glycine) six times for 1 minute, followed by a 30 minute incubation with goat anti-rabbit IgG (conjugated to 10 nm gold, Sigma; diluted 1∶10 in PBS). Grids were then washed in PBS-G (PBS pH7.2, 0.2% glycine) three times for 1 minute. Grids were fixed for 2 minutes (1% paraformaldehyde, 2% glutaraldehyde in PBS), washed in deionised water and negatively stained with 2% methylamine tungstate (Nanoprobes), and observed by transmission electron microscopy in a Tecnai 20 G2 transmission electron microscope (FEI) operated at 200 kV, digital images were collected using an AMT Advantage XR-12 digital camera.

### Cryo-electron microscopy

SARS-CoV was incubated with ACE2 for one hour, and 4 µl samples were applied to glow-discharged holey carbon films supported on 400-mesh copper grids. After blotting immediately for 2–5 seconds with filter paper, grids were plunged into liquid ethane cooled by liquid nitrogen, using a custom built gravity-operated freezing device. Specimens were transferred to a Tecnai 20 G2 transmission electron microscope (FEI) operated at 200 kV, equipped with a Gatan 626.DH low-temperature specimen holder. Observations were made at temperatures of ∼−185°C and images recorded at 29,000× magnification on Kodak SO-163 electron image film at a dose of 10–20 electrons/Å^2^ with an exposure of 1–2 seconds. Film was developed in Kodak D19 for 12 minutes at room temperature, rinsed for 2 minutes in water, and fixed for 10 minutes with Kodak fixer.

### Image processing

The exact magnification in the microscope that SARS-CoV was imaged at was determined to be 29,968× using a calibration grid (Pelco International). Images of SARS-CoV were digitized on a Nikon super coolscan 9000 ED scanner at a pixel size of 2.125Å. Processing was carried out using the EMAN and SPIDER/WEB image processing program packages [35], [45] on SGI Fuel and Tezro workstations running IRIX 6.5, or a Dell PE6850 with 4-way 64-bit Xeon Dual Core 3.6 GHz CPUs and 32GB Ram running Linux (Fedora Core 5). Images were corrected for contrast transfer function (ctf) using the “ctfit” function in the EMAN software package, which estimates defocus and corrects for ctf by phase-flipping. The data set composed of images centred on spikes-ACE2 (n = 11,153; recorded at 2.9–11.9 Å defocus) were processed using SPIDER by the projection matching approach [36] which incorporated three-fold symmetry into the search function and reconstruction procedure. After each refinement cycle the new reference volume was either theresholded or segmented using the “floodfill” function in the SITUS software package [37] to select the spike-ACE2 structure and minimize the influence of neighbouring spikes in the reference structure that was used in the subsequent iteration of the projection match alignment. The initial reference model used in this procedure was the structure of the SARS spike that we previously solved by cryo-electron microscopy [21]. The population of spike images was composed of both side view projections as well as end-on projections, with the latter being easily identified in the images with greater defocus values. These high-defocus/end-on images were essential to eliminate the “missing cone” from the reconstruction. The resolution of the cryo-EM reconstruction was estimated by Fourier shell correlation to be 18.5Å using the 0.5 FSC criteria. The structures of the pre-fusion HR2 domain and the ACE2-receptor-binding domain were docked into the cryo-EM reconstruction using the “floodfill” and “colores” functions in the SITUS software package. The resultant docking generated one distinct location for the ACE2-receptor-binding domain, which placed ACE2 in the additional mass that was not present in the reconstruction of the spike alone. The docking of the entire 2FXP.pdb [2] structure was previously conducted [21].

### Structure visualisation

Three-dimensional cryo-EM reconstructions, and the atomic resolution structures 2AJF.pdb [24], 2FXP.pdb [2], were visualized using UCSF Chimera (Computer Graphics Laboratory, University of California, San Francisco, supported by NIH P41 RR-01081) [46]. The spike component of the spike-viral envelope reconstruction was segmented from the entire reconstruction using the “floodfill” algorithm in the SITUS software package. Movie S1 of the rotating SARS spike-ACE2 complex was generated on a Silicon Graphics workstation using UCSF Chimera, and the “mediarecorder” and “mediaconvert” software provided with the Silicon Graphics IRIX 6.5 operating system. Movie S2 showing the morphing of the spike between the SARS spike and SARS spike-ACE2 complex was generated using the UCSF Chimera software package with the “morph map” morphing tool plug-in. Both reconstructions were masked with a cylindrical mask, which when applied masked the peripheral boundaries of the SARS-CoV envelope in both reconstructions.

## Supporting Information

Movie S1 Three-dimensional reconstruction of the of the SARS spike-ACE2 complex. This movie supplements Figs. 1, 4 showing both the reconstruction of the spike-ACE2 complex as well as the location of the docked atomic structures. The movie starts showing the spike-ACE2 complex viewed from an end-on perspective. The reconstruction then tilts 90o, followed by a 120o rotation along the three-fold axis of symmetry of the spike. In the second section of the movie the cryo-EM reconstruction becomes transparent revealing the location of the docked atomic structures, followed by a final rotation of 90o back to the initial end-on perspective. The following color scheme was used: cryo-EM surface; ACE2, violet; spike, green; stalk, blue; envelope, beige; nucleocapsid, red. Atomic Structures: PDB ID code, 2AJF, (SARS spike receptor-binding domain, red; ACE2, white; ACE2 C-terminus, blue); and PDB ID code, 2FXP, HR2 fusion core, yellow.
(7.24 MB MOV)

Movie S2 Three-dimensional morphing of the SARS spike, and ACE2 bound structures. This movie supplements Figs. 1, 3 showing the putative structural re-arrangement of the spike as it binds to ACE2. The movie starts showing the SARS spike and then gradually morphs to the SARS spike-ACE2 complex, and back to the SARS spike. The morphing reconstruction is shown from the side and end-on perspectives, and the mass attributed to the nucleocapsid is presented without morphing. As one watches the move play back and forth (this can be done by selecting “loop” in a QuickTime Player) one can see the spike twist by ∼5o when viewed from the end-on perspective, and the spike becomes squatter by ∼10 Å when viewed from the side perspective. The following color scheme was used: cryo-EM surface; ACE2, violet; spike, green; stalk, blue; envelope, beige; nucleocapsid, red.
(3.31 MB MOV)
